# From constraints to innovations: a student agency typology and its implications for pedagogical change

**DOI:** 10.3389/fpsyg.2026.1710342

**Published:** 2026-02-17

**Authors:** Wenjun Su, Haisheng Li

**Affiliations:** Institute of Higher Education, East China Normal University, Shanghai, China

**Keywords:** goal orientation, institutional structure, learning behavior, qualitative research, student agency

## Abstract

This qualitative study investigates how Chinese undergraduates navigate the rigid, credential-driven framework of contemporary higher education by examining the interplay between institutional structure and individual agency. Through in-depth interviews with 25 students and a critical typological analysis, this study develops a typology of situated student learning behaviors, grounded in the dimensions of goal orientation (mastery vs. performance) and proactivity (active vs. passive). Four patterns are identified: Active Learning-Oriented, Active Performance-Oriented, Passive Learning-Oriented, and Passive Performance-Oriented. The analysis reveals that students tactically employ a spectrum of strategies—from proactive knowledge pursuit to ritualized compliance—often shifting across contexts. The findings demonstrate how institutional designs centered on performativity and audit culture can incentivize performative labor and educational involution (neijuan), thereby constraining authentic epistemic engagement. The study challenges monolithic views of student passivity and underscores that fostering more learning-oriented agency requires reforms to the underlying institutional logics and reward systems, rather than focusing solely on pedagogical techniques. The typology provides an analytical framework for critically examining performative systems in mass higher education.

## 1 Introduction

In contemporary higher education systems, standardization mechanisms such as GPA calculations, credit requirements, and scholarship thresholds have become key instruments for managing student progress. Scholars theorize this trend as part of a broader shift towards “learning architectures” that prioritize auditable outcomes (Tummons, 2014). While ostensibly ensuring fairness and accountability, these mechanisms are increasingly criticized for privileging quantifiable metrics over meaningful educational engagement and the broader purposes of education (Biesta, 2009, 2020; Tomlinson and Watermeyer, 2022). This critique is central to analyses of the “audit culture” (Shore and Wright, 2015) or “performativity” (Ball, 2003) now prevalent in universities. Thus, a critical question emerges: What happens when the pursuit of credentials eclipses the pursuit of knowledge? This question sits at the heart of mounting global concerns about the rise of performativity in universities and its effects on students’ sense of purpose, autonomy, and learning.

These tensions are particularly visible in East Asian systems such as China’s, where institutional rigidity is pronounced. Students face limited freedom in selecting or changing majors, and academic incentives are tightly bound to measurable outputs. Yet within such uniform systems, variation persists. Some students proactively align their choices with intrinsic interests, while others follow requirements with minimal engagement. These divergent responses—which can include navigating coursework with ingrained habits or strategically expanding social networks to decode institutional expectations (Xie and Dou, 2025)—collectively challenge the view of students as passive recipients of institutional control. Instead, they can be understood as agentic actors who tactically navigate and, at times, subvert structural constraints through processes akin to strategic “figuration work” within bureaucratic environments (Nielsen, 2015). Understanding this agency becomes particularly urgent in contexts shaped by massification, competitive audit cultures, and global discourses of employability.

While scholars have critiqued the global turn toward credentialism and performance metrics (Ball, 2003; Biesta, 2009, 2020), there remains limited understanding of how students in highly structured systems develop situated strategies in response. How does agency manifest when institutional parameters tightly regulate academic life? This study addresses that question by examining how Chinese undergraduates negotiate the tension between credentialist demands and epistemic desires. Through qualitative interviews, we construct a four-part typology of student learning behaviors, offering conceptual tools to better understand the everyday negotiations between systemic constraint and student agency. This study examines how national credential-driven policies (e.g., Gaokao score lock-ins, GPA-centric funding) shape student agency, to inform for rethinking standardization in massified higher education systems.

## 2 Theoretical framework and literature review

To theorize the complex interplay between institutional structures and individual action in contemporary Chinese higher education, this study integrates complementary sociological and psychological perspectives. We first outline the macro-structural context of performativity and institutional logics that shape the educational field. We then introduce the mid-level concepts of goal orientation and tactical agency, which provide lenses for analyzing students’ motivated and strategic responses within these constraints. This integrated framework addresses gaps in a literature that has often critiqued structural pressures without fully theorizing the spectrum of situated student behaviors they elicit, particularly in non-Western, credential-centric contexts like China.

### 2.1 The structural context: performativity, institutional logics, and constraint

Global higher education has been significantly reshaped by the ascendancy of market-oriented institutional logics that privilege competitiveness, measurable outputs, and employability (Cai and Mountford, 2022; Tomlinson and Watermeyer, 2022; Ball, 2012). This framework has been productively applied to analyze diverse educational practices, from community engagement to student learning (Taylor and Kahlke, 2017). To understand the penetration of such logics, one must trace the foundational critique of “academic capitalism,” which systematically analyzes how market and managerial principles have reconfigured academic work, values, and the very conception of knowledge (Slaughter and Rhoades, 2004). A key mechanism of this logic is performativity—a regime of control that rewards the simulation of desired outputs (e.g., high grades, graduate employment rates) often at the expense of substantive processes like deep learning or critical inquiry (Ball, 2003; Tummons, 2014). This fosters an “audit culture” where value is conflated with what is measured and compared (Shore and Wright, 2015).

In China, these global trends intersect with a historically examination-oriented system, producing a uniquely rigid and high-stakes environment. National policies like Gaokao score-based major allocation and university-level GPA-centric scholarship systems materialize a credentialist logic, tightly coupling a student’s academic trajectory with quantifiable performance (Tomlinson and Watermeyer, 2022). This environment can induce a state of educational involution (neijuan), where intensifying competition yields diminishing returns in genuine learning or development, confining students to ritualistic, performative labor and associated psychological strain (He and Liu, 2025; Liu et al., 2024; Yi et al., 2022). This macro-structural backdrop forms the essential context for understanding the pressures students navigate.

### 2.2 Student agency as goal-oriented and tactical practice

Within these constraints, students are not passive but active agents. To capture the dual dimensions of their agency—the motivational “why” and the behavioral “how”—we integrate two mid-level frameworks.

First, goal orientation theory (Dweck, 1986; Elliot and McGregor, 2001) distinguishes between mastery goals (focus on learning, understanding, and competence development) and performance goals (focus on demonstrating competence relative to others and obtaining favorable judgments). In performative systems, these orientations are not merely personal traits but strategic adaptations: a predominant mastery orientation can be an expression of epistemic agency, while a performance orientation often signifies an alignment with institutional reward structures (Senko, 2016). This framework helps categorize the motivational drivers behind student behavior.

Second, to understand how these goals are enacted within rigid structures, we draw on theories of tactical agency and everyday resistance (de Certeau, 1984). Students, lacking the power to overtly change institutional rules, engage in “tactics”—a concept de Certeau (1984) termed the “art of the weak.” These are subtle, adaptive practices of appropriation, circumvention, and selective compliance that allow individuals to create or maintain spaces of autonomy and serve personal ends within systems not of their making (Nielsen, 2015; Scott, 1990). This perspective reconceptualizes student behavior from a binary of compliance/deviance to a strategic negotiation, encompassing actions as varied as auditing interesting courses outside one’s major or tailoring effort to graded components. It thus addresses the critical interplay between institutional structures and student agency, a central concern in understanding student engagement and success (Kahu and Nelson, 2018).

### 2.3 Synthesizing the framework and identifying the gap

While the critical literature robustly details structural constraints (Ball, 2003; Marginson, 2014; Tomlinson and Watermeyer, 2022), and psychological research maps motivational profiles (Dweck, 1986; Senko, 2016), significant gaps remain. Few studies systematically examine how these macro and micro forces combine to produce recognizable, situated patterns of student behavior within highly structured systems. Research on student agency in China, while growing, often focuses on broad adaptations or psychological well-being (Xie and Dou, 2025), lacking a fine-grained typological analysis that connects specific institutional logics (e.g., credentialism, involutionary competition) to repertoires of goal-directed tactics.

This study addresses this gap by developing a grounded typology. It positions students as tactical agents whose goal orientations (mastery vs. performance) and mode of engagement (active vs. passive) intersect to form distinct behavioral patterns within the performative architecture of Chinese higher education. This integrated framework offers a nuanced lens for analyzing the varied ways students navigate, resist, and sometimes surrender to the credentialing paradox (Table 1).

**Table 1:** An analytical framework of student agency and structure.

Analytical level	Core concept	Operational meaning in this study	Key references
Macro-structural	Performativity and credentialist logic	The dominant institutional logic that prioritizes measurable outputs (grades, credentials) and structures rewards and competition.	Ball (2003); Cai and Mountford (2022); Tomlinson and Watermeyer (2022)
	Involution (Neijuan)	A resultant state of hyper-competition within this logic, where increased effort yields diminishing returns in authentic learning.	He and Liu (2025); Liu et al. (2024)
Agentic-practice (meso-level)	Goal orientation	Students’ primary motivational focus: mastery (epistemic growth) vs. performance (external validation) within the system.	Elliot and McGregor (2001); Senko (2016)
	Tactical agency	Students’ situated, strategic practices to navigate, appropriate, or subtly resist the above structural pressures.	de Certeau (1984); Nielsen (2015)

## 3 Methodology

This study employs a critical typological analysis (Kluge, 2000) rooted in interpretive methodology, foregrounding students’ subjective experiences and situated agency within institutional structures. Drawing on de Certeau’s (1984) notion of “tactical agency,” our approach treats students not merely as informants but as theorists of their own lives—active participants who navigate higher education systems shaped by standardization and credentialism.

### 3.1 Participants and data collection

A purposive sample of 25 undergraduate students was recruited from a large, research-intensive university in Eastern China to capture a range of experiences within the credential-driven system. Participants represented all four undergraduate years and spanned three broad disciplinary clusters: Sciences (*n* = 9), Social Sciences (*n* = 8), and Humanities (*n* = 8). Semi-structured interviews, conducted in Mandarin and lasting 45–75 min, served as the primary data source. The interview protocol was designed to elicit rich, contextual narratives about academic decision-making and was informed by the theoretical framework outlined in Section 2. Key question areas included: (1) motivations and strategies for selecting courses and majors; (2) approaches to classroom participation and assignment completion; (3) perceptions of grading, assessment, and institutional reward structures (e.g., scholarships); and (4) experiences with specific institutional rules and pressures (e.g., major transfer policies, GPA thresholds). All interviews were audio-recorded, transcribed verbatim, and anonymized.

### 3.2 Data analysis and typology construction

The analysis followed a multi-phase, iterative process to develop a grounded typology of student learning behaviors, ensuring a transparent chain of evidence from raw data to theoretical categories (Saldaña, 2021).

Phase 1: To ensure coding reliability, this study employed an iterative coding process. All transcripts were first coded by the primary researcher. To verify consistency, a second round of recoding was conducted on all transcripts after a two-week interval. Throughout this process, analytical memos were kept to iteratively refine the emerging themes. Initial codes captured concrete descriptions of behaviors and decisions, such as “choosing a major based on personal interest,” “selecting courses perceived as easy for high grades,” or “complying with teacher authority despite personal preference.

Phase 2: *Contextual embedding*. These coded behaviors were then analyzed in relation to the institutional contexts described by students. Through axial coding, we explicitly linked individual actions to perceived structural conditions, such as high-stakes exam formats, rigid major transfer rules, and scholarship criteria tied to GPA rankings. This step ensured that behavioral motifs were understood as responses to specific institutional pressures.

Phase 3: *Constructive abstraction*. Through constant comparative analysis of the codes and their contexts, two fundamental, binary dimensions consistently emerged as organizing principles for the observed behaviors: Goal Orientation (Mastery vs. Performance) and Proactivity (Active vs. Passive). These dimensions form the axes of the resulting typology.

Phase 4: *Typological synthesis and empirical grounding*. A 2 × 2 matrix was constructed based on the intersecting dimensions (Table 2). Each quadrant defined a distinct behavioral type. To ground these abstract types in empirical data, we returned to the coded transcripts, identifying representative narrative excerpts that concretely demonstrated the logic and context of each behavioral pattern. It is crucial to note that the typology classifies behavioral episodes, not students as whole individuals. A single student’s interview often contained accounts of different strategies across varying academic situations (e.g., pursuing a personal research interest vs. preparing for a required core course exam). Therefore, a participant’s narrative could contribute data to more than one behavioral category. This approach accurately reflects the situational and tactical nature of agency, acknowledging that students deploy different strategies in response to varying pressures (Cantwell et al., 2018). For instance, student S12’s narrative included both an account of choosing a major based on personal interest, coded as an Active Learning-Oriented episode, and an explanation of selecting a major to maximize the utility of Gaokao scores, coded as an Active Performance-Oriented episode. This concretely illustrates that our typology classifies contextual behaviors, not students as fixed types.

**Table 2:** A typology of student learning behaviors.

Agency mode (Active/Passive)	Learning goal orientation	Performance goal orientation
Active	“Choose a major that interests me”	“I should choose a major with a higher admission score so as not to waste my college entrance exam score.”
	“Learning makes me inexplicably happy.”	
Passive	“Complete the task first, then consider my interests.”	“Everyone is involuting, so I have no choice but to join in.”

## 4 Results

Following the analytical logic of classifying behavioral episodes (see Section 4.2), the distribution of the coded episodes across the four types was as follows: Active Learning-Oriented (*n* = 32 episodes), Active Performance-Oriented (*n* = 41 episodes), Passive Learning-Oriented (*n* = 28 episodes), and Passive Performance-Oriented (*n* = 39 episodes). It is important to reiterate that these counts refer to instances of behavior, not unique students; all 25 participants contributed episodes to at least two different types, illustrating the contextual nature of their agency.

### 4.1 Active learning-oriented behavior

Students whose behaviors were categorized as Active Learning-Oriented consistently described pursuing knowledge and intellectual challenge as their primary aim, even within the constraints of institutional structures. In their accounts, the process of learning was consistently prioritized over and described as more fulfilling than the pursuit of extrinsic rewards, such as grades or credentials.

For instance, when choosing a major, these students tend to follow their personal interests rather than external pressures. One student described,

“The more I studied here, the more I realized that this wasn’t what I wanted, that it wasn’t my favorite major. So I decided to switch majors and ended up studying physics, which was what I had always wanted to study” (S11).

Even when initially guided by pragmatic considerations such as course difficulty or advice from peers, they often recalibrate their decisions when they realize such courses lack intellectual engagement. As another student shared,

“After that, I realized that I shouldn’t choose something that doesn’t teach me anything. Choosing what interests me most has been quite rewarding” (S12).

A common expression among these students is the idea of “challenging oneself,” which encapsulates not only their desire to meet academic challenges but also their recognition of intellectual growth as a deeply rewarding experience. This mindset drives them to seek learning opportunities that not only satisfy their personal curiosity but also enhance their academic development. For example, in the classroom, although many students choose not to participate for reasons such as personality or perceived lack of expertise, those with an active learning orientation push beyond these limitations. As one student explained,

“Whenever our group asks, ‘Who wants to answer the question?’, I am always the first to say, ‘I will.’ They are reluctant to answer questions, but I am eager to challenge myself” (S13).

For students in this category, learning itself transcends transactional objectives such as grades. The pursuit of knowledge is a source of non-instrumental fulfillment, offering a form of intrinsic joy. As one student put it,

“Studying is quite enjoyable. Whether it’s a course or a lecture, I prefer those with a stronger academic focus. After listening, I feel like I’ve gained a lot, and I feel inexplicably happy” (S14).

Their narratives collectively depict a pattern of continuously seeking challenging learning opportunities aligned with personal interest, even when institutional incentives are limited.

### 4.2 Active performance-oriented behavior

Students whose behavioral episodes were classified as Active Performance-Oriented primarily focused their accounts on optimizing measurable academic outcomes, such as grades and GPA, to secure associated benefits like scholarships or graduate school admission.

This strategic approach was evident in major academic decisions. For example, one student shared,

“Because I didn’t perform well on the college entrance exam at the time, I wanted to use my scores to get into a relatively good major or a relatively good school so as not to waste those scores” (S12).

Many students admitted to competitive majors with high entrance scores hold a similar perspective. As one student stated,

“Since I originally got in with high scores, changing majors would be a waste of my college entrance exam scores, so it doesn’t seem worth it” (S18).

In their accounts, these students frequently described a decision to remain in their initial major, even when expressing limited personal interest in the field. Their justifications for this choice centered on two themes mentioned in interviews: first, a desire not to devalue their college entrance exam achievement; and second, the perceived career or status advantages associated with the original, highly competitive major.

Consequently, these students also described carefully weighing various factors during course selection, including perceived course difficulty, instructor reputation, and the potential impact on their GPA. One student described this process:

“First, check whether the teacher gives high marks. If you are unsure, ask them. For general education courses, getting high marks will not only earn you credits, but also raise your GPA” (S18).

This calculus often led them to choose courses perceived as more straightforward pathways to high grades.

For these students, final exams were frequently described as the most critical determinant of performance. They often perceived these high-stakes exams as testing a narrow range of textbook-based content, which in turn shaped their preparation strategies towards focusing on predictable testable material. One student reflected on this strategic approach:

“Although I don’t study very hard normally, I pray to the gods before exams that the questions I guess will be on the test. If I’m lucky, I’ll save a lot of time compared to others and get a higher score” (S23).

The accounts of these students reveal a pattern of calculated, strategic investment aimed at maximizing returns within the evaluation system.

### 4.3 Passive learning-oriented behavior

Students whose behaviors fell into the Passive Learning-Oriented type described navigating their academic paths by first fulfilling institutional requirements, while seeking limited, sanctioned opportunities to engage with content aligned with personal academic interests.

One student described balancing compliance with the pursuit of better instruction:

“My homeroom teacher does our culture class, but he’s not that interesting. The teacher next door is way better—I often slip into their lectures after mine finishes. But I’d never skip my homeroom teacher’s class. Seriously, I wouldn’t dare” (S12).

(The term “homeroom teacher” here refers to a role in Chinese universities that combines teaching with student management responsibilities.) This pattern of compliant exploration was also evident in other accounts. One student, for instance, mentioned completing all mandatory problem sets first before allowing time to read historical articles related to the course topic, which they found more intriguing (S07).

Another example illustrates this dynamic further:

“My English teacher’s fine, but the other class has this returnee professor—his pronunciation’s perfect, like a native speaker’s. I love listening to him, but I can only catch his lectures when I’m free. Got my own language classes and major courses to attend, you know?” (S14).

For instance, she described occasionally attending the first half of the returnee professor’s lecture during a gap in her own schedule, even if it meant leaving early to get to her next required class. Here, the student’s access to preferred learning content is described as constrained by the fixed institutional schedule.

Their behavior is characterized by seeking a balance between strict adherence to institutional frameworks and the fulfillment of personal intellectual curiosity.

### 4.4 Passive performance-oriented behavior

The behavioral pattern classified as Passive Performance-Oriented was characterized in student accounts by a focus on ritualized compliance with the formal requirements of the assessment system, where effort was directed towards meeting metrics rather than deep engagement.

Students frequently described how specific institutional rules shaped their behavior in calculable ways. For example, one student explained the direct link between grading rules and classroom participation:

“I’m passive by nature, but if participation counts toward grades, we’re all forced to speak. No points? No engagement” (S9).

In such instances, participation itself was often described as a transactional activity aimed at securing fractional credit points rather than intellectual exchange.

This focus on meeting formal metrics extended to assignment completion. Many students reported adapting their work to perceived grading rubrics rather than personal understanding. One student shared a common strategy:

“Writing 2 pages suffices for learning, but scoring 90+ requires 10+ pages. We copy formats, not ideas—this is involution (neijuan)” (S25).

Several other participants similarly described “padding” essays with redundant content or directly replicating model answers, identifying these as widespread practices necessary for high grades but disconnected from learning.

Students often reflected on the consequences of this engagement pattern, noting a significant expenditure of time and effort on tasks they perceived as substantively “empty” or “simulated.” This frequently led to feelings of inefficiency and a lack of genuine intellectual progress. As one student summarized, much time was spent “jumping through hoops” that did not contribute to actual knowledge growth.

This pattern of behavior is defined by a ritualized fulfillment of external assessment rules, where the primary goal is to meet the metric itself.

Table 3 synthesizes the core behavioral strategies that characterize each of the four typologies presented above.

**Table 3:** Behavioral strategies across the four typologies.

Behavior type	Major selection	Goal pursuit	Institutional negotiation
Active learning	Interest-driven defiance	Knowledge for understanding	Pursuing interest-aligned paths despite credentialing pressures
Active performance	ROI optimization	Credentials for advancement	Strategically selecting courses and tasks to maximize scores/GPA
Passive learning	Requirement-first compliance	Covert curiosity	Fulfilling requirements while seeking limited interest-based engagement
Passive performance	Path-dependent inertia	Ritualized compliance	Completing tasks to the minimal required standard for credit

## 5 Discussion

This discussion synthesizes the empirical typology with broader debates on student agency and performativity in higher education, theorizing the observed behaviors as situated responses to institutional structures. It proceeds in four parts. First, we conceptualize the typology as a form of situated praxis, emphasizing the tactical nature of student agency (see Section 5.1). Second, we analyze students’ adaptation to the economy of symbolic resources through performative labor (see Section 5.2). Third, we trace these behaviors to a core structural contradiction between educational ideals and metric-driven governance (see Section 5.3). Finally, we integrate these insights to show how differentiated agency emerges as patterned responses to institutional imperatives (see Section 5.4).

### 5.1 Typology as situated praxis

The four-fold typology developed in this study challenges a binary understanding of students as either compliant or resistant. It conceptualizes student agency as a situated praxis—a repertoire of strategic behaviors dynamically shaped by the interaction between personal goal orientations (mastery vs. performance) and perceived institutional constraints (Biesta, 2015; Eteläpelto et al., 2013). Here, we operationalize agency as a core and dynamic component of student engagement (Reeve and Tseng, 2011), observable through these strategic behaviors. Our findings reveal that within the rigid architecture of Chinese higher education, students are not passive recipients but active negotiators who employ a spectrum of tactics, engaging in what can be understood as “tactical agency” within structured systems (de Certeau, 1984).

The Epistemic Architects (Active Learning-Oriented) construct individualized pathways by aligning actions with intrinsic interests, often strategically circumventing credentialing pressures. In contrast, the Credential Strategists (Active Performance-Oriented) engage in a calculated process of optimization within the system, aiming to maximize institutional outputs. The Covert Learners (Passive Learning-Oriented) navigate by fulfilling requirements first while seeking pockets of authentic engagement, embodying a form of constrained agency. Finally, the Ritualistic Compliers (Passive Performance-Oriented) have largely internalized the logic of performativity. Theoretically, their behavior exemplifies “surface learning”—investing effort in symbolic compliance aimed at meeting external requirements, with minimal genuine epistemic engagement (Dolmans et al., 2016). Together, these four types illustrate the contingent and tactical nature of student agency within the system.

### 5.2 Resource adaptation and symbolic effort

Within credential-driven higher education systems, students’ everyday learning practices are strongly shaped by their positioning within an economy of symbolic and material resources, in which GPA operates as the primary currency for accessing scholarships and future opportunities. As students internalize this competitive logic, they increasingly develop strategic forms of adaptation aimed at optimizing measurable outcomes rather than deep engagement.

The most common strategies observed—course selection for GPA inflation, emulation of high-performers’ routines, and the inflationary padding of assignments—exemplify what we term performative labor. This represents a distinct shift in effort from deep cognitive investment to the symbolic display of effort, aimed at meeting audit metrics rather than achieving understanding. The goal shifts from producing what suffices for learning to what merely “scores 90+”— a vivid illustration of the displacement of epistemic by performative ends.

In the Chinese context, this pervasive pattern of performative labor aligns with and concretizes the broader phenomenon of educational involution (neijuan) (Geertz, 1963; Yu, 2025). It manifests as a condition where intensifying effort (e.g., writing 10-page essays) yields diminishing returns in genuine intellectual growth, trapping students in ritualistic cycles. For many, as reported in the interviews, the very meaning of learning became synonymous with credential output—“getting a scholarship” or “staying above the GPA line.” This reflects a profound shift wherein students are, in effect, participating in a high-stakes signaling economy (Tomlinson and Watermeyer, 2022), where education is pursued for its exchange value rather than its transformative potential. This instrumental engagement, often termed “performative labor,” is not only cognitively hollow but can also incur significant emotional costs (Gretzky and Lerner, 2021). This outcome starkly illustrates the “self-formation paradox” (Marginson, 2014) under audit cultures, where systems designed to foster autonomous graduates instead incentivize the strategic accumulation of credentials.

### 5.3 The core structural contradiction

Patterns of student adaptation point to a deeper structural contradiction embedded in contemporary higher education governance. While universities formally endorse educational ideals such as autonomous learning, critical inquiry, and intellectual development, student success is predominantly evaluated through standardized and comparative metrics, most notably GPA and credential accumulation. This misalignment generates persistent tension, shaping not only what forms of engagement are rewarded, but also which forms of agency become viable within the system.

At its core, the contradiction revealed by our typology reflects a deeper conflict between the market-driven logic of performativity and the cognitive or pedagogical logics traditionally central to university education (Cai and Mountford, 2022). This tension embodies the long-standing institutional clash between the “academic logic” of knowledge cultivation and the “market logic” of commodification and performance measurement, a central thesis of the academic capitalism framework (Slaughter and Rhoades, 2004; Schulze-Cleven and Olson, 2017). Recent scholarship conceptualizes this market logic as evolving from commodification to ‘assetization,’ where universities, through digital standardization and intellectual property restructuring, transform pedagogy itself into a controlled asset whose value is tied to expectations of future revenue rather than immediate educational substance (Ayers, 2025). This process, as captured in the strategic adaptations of our student typology, concretizes how performative logic systematically constrains the space for pedagogical autonomy and epistemic exploration.

### 5.4 Student agency and structural imperatives

When institutional reward structures consistently privilege measurable performance over epistemic engagement, student agency does not disappear but becomes differentiated in patterned ways. Under these structural imperatives, students adopt distinct strategic orientations that reflect varying alignments between personal goal orientations and institutional demands. The typology developed in this study captures these differentiated responses, illustrating how student agency is reconfigured into a spectrum of situated adaptations within credential-driven systems.

Specifically, Epistemic Architects (Active Learning-Oriented) represent a minority who manage, often with difficulty, to align intrinsic mastery goals with the institutional demand for performance. In contrast, Credential Strategists (Active Performance-Oriented) respond with instrumental rationality, strategically aligning their efforts with the system’s measurable outputs, sometimes at the expense of deeper engagement. Covert Learners (Passive Learning-Oriented) navigate this tension through a compromise: they fulfill mandatory requirements first, while cautiously seeking supplementary opportunities for authentic intellectual engagement within the rigid system. For Ritualistic Compliers (Passive Performance-Oriented), the contradiction results in a disengaged, habitual form of participation where compliance is an end in itself, devoid of aspirational or intellectual content.

This pattern of responses underscores a critical theoretical point: student behavior is profoundly shaped by the design of institutional incentives. Grounded in goal orientation theory, individual adoption of mastery or performance goals is highly sensitive to contextual cues about what is valued (Dweck, 1986; Elliot and McGregor, 2001). The prevalence of performance-oriented behaviors in our study is, therefore, not primarily a reflection of student character, but a rational adaptation. This adaptation must be understood at a systemic level: when the institutional “rules of the game” so clearly prioritize and reward measurable outputs, a shift towards performative engagement is the predictable result.

Therefore, reforming these patterns necessitates a critical redesign of the underlying reward structures, not merely pedagogical appeals for change.

## 6 Conclusion

This study has developed a grounded typology of student learning behaviors to elucidate how Chinese undergraduates negotiate the rigid, credential-driven framework of contemporary higher education. The four identified types—Active Learning-Oriented (Epistemic Architects), Active Performance-Oriented (Credential Strategists), Passive Learning-Oriented (Covert Learners), and Passive Performance-Oriented (Ritualistic Compliers)—demonstrate that students are not merely passive. They actively engage in calculated navigation, strategic optimization, constrained compromise, or ritualistic compliance in response to systemic pressures. The widespread adoption of performance-oriented strategies and the observed patterns of performative labor and educational involution are not merely student choices but rational adaptations to an environment where success is narrowly defined by measurable outputs like GPA and credentials.

The central contribution of this study is to reframe the understanding of student engagement within highly structured systems. It highlights a fundamental structural contradiction between the stated aim of fostering critical, autonomous learners and the institutional reliance on standardized performance metrics. This contradiction channels student agency toward instrumental and ritualistic forms of engagement. Consequently, the behavioral patterns documented are best understood as outcomes of the existing institutional design.

While derived from the context of a Chinese research-intensive university, the core dimensions of the typology—goal orientation and proactivity—offer a transferable analytical framework. It provides a tool for researchers and educators in diverse contexts to diagnose the spectrum of student agency within their own performative systems. Ultimately, this study argues that fostering more authentic, learning-oriented engagement requires moving beyond critiquing student behavior to rethinking the architectural logic of incentives and assessments that shape it.

## 7 Implications for practice

The identified typology reveals systemic tensions between credentialing mechanisms and authentic learning. To reconcile institutional accountability with student agency, we propose three evidence-informed pathways for reform:

### 7.1 Restructuring institutional frameworks

To dismantle the rigid credentialist logic, institutions must undertake the following three structural reforms:

First, diversify academic pathways by expanding minors, dual-degree programs, and interdisciplinary curricula. Such flexibility, a key component of responsive systems (Barua and Lockee, 2024; Jonker et al., 2020), enables personalized learning journeys within the formal curriculum.

Second, redesign assessment ecosystems by reducing high-stakes exams, integrating formative evaluations (e.g., portfolios), and adopting multi-dimensional scholarship criteria. Research supports formative assessments for deepening engagement (Kulasegaram and Rangachari, 2018).

Third, embed developmental advising with trained mentors from the first year, focusing on three core tasks: (1) mapping students’ intrinsic interests to institutional opportunities through structured exploration; (2) co-creating learning goals beyond credential accumulation via reflective dialogue; and (3) navigating structural constraints through strategic agency. This model is designed to foster student agency and metacognitive growth (Morris et al., 2021).

### 7.2 Reimagining pedagogical contracts

Faculty must shift from instruction to co-construction by designing classrooms as participatory inquiry zones through three strategic actions.

First, negotiate syllabi with students to incorporate self-proposed topics—a practice shown to increase ownership and belonging (Oruç, 2024). Second, replace monologic lectures with Socratic seminars and dialogic methods, which are effective in fostering deeper cognitive engagement and critical thinking (Cui and Teo, 2021). Third, assess learning processes through reflective journals and portfolios alongside final products, thereby valuing intellectual development beyond tangible outcomes.

Concurrently, educators must design assessments that explicitly disrupt performativity traps and resist instrumental “gaming.” Key tactics include: critiquing credential-gaming behaviors in class discussions; capping assignment lengths to deter ritualized padding; diversifying task formats (e.g., podcasts, infographics); and decoupling participation grades from mere verbal quantity. Such alternative methods better capture complex learning and reduce strategic compliance (Guha and Roy, 2025).

### 7.3 Cultivating critical agency

Advisors should reframe their role from transactional informants to transformative mentors who cultivate students’ metacognitive capacities. This shift requires three key practices.

First, mentors should guide students to articulate personal learning purposes that transcend employability, connecting daily tasks to broader goals of ethical reasoning, civic engagement, or intellectual growth. The framework of fostering “self-authorship” (Magolda, 2023) and the strategic use of structured reflection (Jackson and Trede, 2020) are central to this purpose-driven mentoring.

Second, mentors must equip students to identify and navigate structural barriers by making the “hidden curriculum” explicit. Targeted mentoring is crucial for helping students decode and strategically navigate these unwritten rules (Haeger et al., 2018).

Third, effective mentoring supports aligning daily academic choices with long-term self-formation goals through co-created strategic plans. Research shows that programs integrating goal-setting with structured exploration enhance students’ academic adjustment and proactive agency (Sampaio et al., 2025; Santilli et al., 2020).

Integrating these mentoring practices with the institutional and pedagogical reforms outlined above enables a coherent system that fosters the critical agency necessary for authentic, learning-oriented engagement.

## 8 Limitations and future research

This study has several limitations that point to valuable avenues for future work. First, the typology is derived from interviews at a single research-intensive university in China. Its transferability to other institutional contexts (e.g., teaching-focused or vocational colleges) and cultural settings requires verification through broader comparative sampling.

Second, while the typology captures the situational and tactical nature of student agency, its cross-sectional design cannot trace how students’ strategic orientations evolve over their extended academic journey. Future longitudinal research is needed to map these developmental trajectories and understand shifts in agency over time.

Third, a deeper exploration of contextual moderators is warranted. Future research should investigate how factors such as disciplinary cultures, peer networks, and socioeconomic backgrounds shape the adoption of particular behavioral strategies, thereby clarifying the boundary conditions and moderating mechanisms of our typology.

Finally, this study establishes behavioral categories but does not empirically link them to long-term developmental outcomes, such as critical thinking skills, well-being, or career adaptability. Future work should investigate these links and, most importantly, empirically test the institutional and pedagogical reforms proposed in Section 7. Ultimately, without such empirical validation and a concomitant redesign of the policy architectures that privilege measurable outcomes, the credentialing paradox identified in this study is likely to persist.

## Data Availability

The original contributions presented in the study are included in the article/supplementary material, further inquiries can be directed to the corresponding author.
